# Patient Contentment Regarding Health Education Services at King Saud Medical City in Riyadh, Saudi Arabia

**DOI:** 10.7759/cureus.66960

**Published:** 2024-08-15

**Authors:** Abdulrahman M Elnasieh, Atheer T Alturki, Razan Alhadlaq, Mohammed Almesned, Akram N Al-Hazm, Hareth Almajid, Waleed Ahmad Alayyafi, Ahoud Saad Alzuwaidi, Mawada A Elnasieh

**Affiliations:** 1 Family Medicine, King Saud Medical City, Riyadh, SAU

**Keywords:** saudi arabia, health education, healthcare quality, primary healthcare services, patient satisfaction

## Abstract

Background: Health education enhances healthcare outcomes and patient satisfaction, and with digitalized methods, it is gaining popularity in high-income nations. Effective education promotes behavioral change, treatment adherence, and overall satisfaction while maintaining interpersonal communication. Despite the strides made in medical advancements for diagnosis and treatment, interpersonal communication remains the primary conduit for information exchange, particularly manifested through health education dialogues between medical practitioners and patients.

Methodology: A cross-sectional study was conducted at King Saud Medical City (KSMC), Riyadh, Saudi Arabia, to assess patient satisfaction with health education services. Data were collected through a structured questionnaire. Data were analyzed by IBM SPSS Statistics for Windows, Version 29 (Released 2021; IBM Corp., Armonk, New York, United States).

Results: This study on patient satisfaction with health education at KSMC in Riyadh included 225 participants, predominantly females (67.6% (n=152), mean age 38.5 years). Diabetes was the most prevalent (21.3%, n=48) among participants. Doctors were the primary source of health advice (46.2%, n=104). Structural aspects received high satisfaction (mean score of 31.8), surpassing healthcare provider delivery (mean score of 24.9) and print materials (mean score of 22.7). Demographically, occupation significantly impacted contentment (p-value=0.002), with students exhibiting the highest scores. Logistic regression highlighted patients' occupation (aOR=1.498) and patients' level of education (aOR=0.420) as predictors of contentment.

Conclusion: This study highlighted high satisfaction with structural aspects of health education. Occupation, particularly among students, significantly impacts contentment. Tailoring education strategies based on occupation and education levels is crucial for improved patient satisfaction.

## Introduction

Health education is acknowledged as a pivotal element in attaining enhanced healthcare results. Greater contentment among patients with the educational services offered is associated with enhancements in health outcomes and the overarching quality of healthcare. Consequently, healthcare providers equipped with an understanding of patients' viewpoints regarding the dispensed health education and the evaluation of their satisfaction contribute to recognizing avenues for refinement, thus bolstering the overall effectiveness of the system. The assessment of patient satisfaction carries significance for physicians, healthcare administrators, and patients alike, ensuring that healthcare standards are achieved and upheld [1].

Health education proves to be a potent method for disseminating knowledge and motivating the populace to adopt healthier lifestyles. This subsequently serves as a preventive measure against diseases and mitigates ensuing complications [1].

Numerous multimedia resources, including web-based educational tools, have become accessible to enhance various aspects of perioperative care. Utilizing these educational formats can assist outpatient centers in enhancing patient satisfaction during outpatient surgical procedures while simultaneously enhancing patient safety [2].

The primary benefit of implementing standardized healthcare practices is that they facilitate a better grasp of patients' conditions and enable the timely monitoring of relevant health indicators. Standardized health education enhances rehabilitation training by conducting meticulous and precise assessments of functions, ultimately leading to improved activities of daily living and, consequently, an overall enhancement in the quality of life [3].

Patient satisfaction plays a significant role in influencing the clinical process and patient outcomes. Numerous studies have indicated that there is a connection between patient satisfaction and improved patient outcomes. Findings from systematic reviews have demonstrated that patient satisfaction is positively linked with safety, clinical effectiveness, adherence to recommended care, and the utilization of screening services. Thus, patient satisfaction has the potential to yield a range of positive outcomes [4].

Contemporary medical care principles like informed consent and shared decision-making hinge on the assumption that patients comprehend the information provided to them regarding their condition and treatment. Patient education materials that surpass the recommended readability level potentially disrupt the informed consent and shared clinical decision-making process. This disconnect might hinder patients' comprehension of these resources and potentially lead to subpar health outcomes and reduced patient satisfaction [5].

Health education is an integral aspect of the responsibilities of medical personnel, as it involves conveying health-related knowledge and techniques for managing diseases to communities, families, and individuals through suitable educational methods. Traditional health education typically involves healthcare staff providing oral instruction or distributing health education materials [6].

Health administrators prioritize the dissemination of high-quality health education information to empower patients to take a more active role in their healthcare, especially when it comes to managing chronic conditions or promoting overall well-being. This approach generally involves patients actively sharing their healthcare priorities and actively participating in the decision-making process [7].

Digitalized educational methods, including smartphone applications, videos, web-based content, and virtual reality, are becoming more widely adopted in patient education across various medical fields. The utilization of immersive patient education formats that align with patients' situational and environmental expectations in diverse settings seems to be an effective approach for reducing anxiety and enhancing patient satisfaction [8].

While digital education has been a part of health education for the past two decades, its technological advancements and widespread adoption have accelerated in recent years, especially in high-income nations. Nevertheless, there exists a global demand for scalable and top-notch education to enhance the skills of healthcare professionals, especially in countries dealing with workforce shortages and a growing burden of chronic illnesses [9].

Presently, health education is acknowledged as a pivotal factor in attaining enhanced healthcare results. Elevated patient contentment with educational services is currently correlated with enhancements in healthcare quality. Patient satisfaction feedback assists healthcare providers in identifying potential zones for enhancement, thereby bolstering the efficacy of healthcare systems. Satisfied patients are inclined to revisit ongoing care, advocate the healthcare facility to others, and cultivate a foundation of trust with their healthcare providers. Such patients are also more prone to adhere to the medical provider's guidance and the recommended treatment [10].

There is a prevailing belief that educational interventions exert a positive influence on behavioral transformation. Research has substantiated that patient education stands as the most efficacious means of imparting knowledge, and facilitating adherence to prescribed treatment regimens. Moreover, this approach promises to be cost-effective and impactful in elevating patient satisfaction [11].

Health education is crucial for improved healthcare outcomes and patient satisfaction. Similarly, Paterick et al. (2017) show that enhanced healthcare outcomes require physicians' dedicated, enthusiastic, and responsive interaction, fostering robust engagement with patients for education [12]. Various methods, including multimedia resources and standardized practices, contribute to enhancing patient understanding and engagement [13]. Patient satisfaction links positively to safety, clinical effectiveness, and adherence to care [14]. The integration of digitalized educational methods is rising, especially in high-income nations, addressing the global demand for scalable healthcare education [15]. Health education drives behavior change, treatment adherence, and patient satisfaction, emphasizing vital interpersonal communication [16].

Hence, this study was conducted to evaluate patient contentment concerning the health education services offered at King Saud Medical City (KSMC), Riyadh, Saudi Arabia, across various clinical care environments, to investigate the correlation between participants' satisfaction levels and their demographic and personal attributes, and enhancing the quality of health education services by offering a contemporary understanding of participant satisfaction based on existing evidence. This will help inform future recommendations for this essential domain.

## Materials and methods

Study design and study setting

This was a self-administered cross-sectional descriptive survey conducted at the outpatient department and staff clinic waiting areas of KSMC.

Study population and eligibility criteria

The study targeted adult patients of both genders attending the outpatient department and staff clinics at KSMC during the study period. Specifically, it included all adult patients visiting these facilities who were willing to participate after receiving counseling and had prior exposure to health education services at KSMC.

However, the study excluded patients visiting the dental, radiology, physical therapy, obstetrics and gynecology, and pediatric departments, and laboratory during the research timeframe. These exclusions were made because these departments are located in different areas of the hospital and have limited and restricted accessibility. Additionally, the study did not involve the relatives accompanying these patients.

Study timeframe 

The study was conducted over two months from November 1, 2023, to December 31, 2023. During the study period, all patients in the study settings who had experience with health education services at KSMC were invited to participate in the study. Enrollment was determined through randomization of the participants, ensuring that all attendees in the study settings had an equal opportunity to be included.

Sampling technique and sample size

The sample sizes of previously published studies with similar aims and populations were reviewed and adopted to support the appropriateness of the current study sample choice. These studies used comparable sample sizes, lending credibility to the decision [10,11]. The sampling method employed was a convenience sample, including all adult patients of both genders attending the outpatient department and staff clinics at the waiting areas of KSMC during the study period.

Study tool and data collection

A validated close-ended structured questionnaire was obtained from a previous study conducted by Asiri et al., in 2013 in Riyadh, Saudi Arabia [10]. The obtained questionnaire was modified and translated into Arabic.

Study variables

Within this study, the dependent variable concerns patient satisfaction with health education services, serving as the central outcome. This facet was detailed in the questionnaire and encompassed levels of contentment related to adequate time allocated for discussions with a trusted health educator and general overall satisfaction. The patient satisfaction questionnaire was developed using a previously established questionnaire from the literature, which focused on the benefits of health educational services and patient satisfaction [10].

Participant responses were outlined in the questionnaire. Conversely, the independent variable encompassed a range of factors hypothesized to affect and interrelate with the dependent variable, essentially contributing to its manifestation or impact. These factors encompass patients' demographic and personal characteristics, as well as the structure, processes, and outcomes inherent in health education services.

Statistical analysis

A comprehensive statistical analysis was conducted on the dataset, encompassing both descriptive and inferential methodologies. First, a descriptive analysis was conducted to summarize the demographic characteristics of the participants, which include age, gender, and other features' frequency and percentages. This provides an overview of the study population. Subsequently, inferential analyses such as ANOVA (for more than two groups) were employed to examine contentment score differences between different occupations, and the non-parametric Friedman Test was used to find out the difference between mean scores of different components of services. Multivariate logistic regression analysis was conducted to find out the factors for high contentment among participants. Statistical significance was established at a p-value of 0.05 or lower and a 95% confidence interval. All statistical analyses were executed using IBM SPSS Statistics for Windows, Version 29 (Released 2021; IBM Corp., Armonk, New York, United States).

Ethical consideration

The proposal was approved by the Institutional Review Board (IRB) of the KSMC Research and Innovation Center via proposal reference number H1RI-24-Oct23-03, dated October 24, 2023. The researchers guaranteed that respondents' identities remained anonymous, the collected data remained confidential, and consents were signed by the participants.

## Results

The study included 225 participants. The majority of participants were females (67.6%, n=152), with a mean age of 38.5 years (SD=15.5). Occupation-wise, a notable proportion were not employed or retired (25.3%, n=57), followed by housewives (21.3%, n=48). Regarding marital status, a majority of participants were married (62.7%, n=141). Educationally, a substantial number had completed high school (36.9%, n=83) or university (39.1%, n=88) (Table 1).

**Table 1:** Sociodemographic data for the participants (n=225) The data is presented in frequency (n) and percentage (%). Except for age where data is presented as mean and SD.

Demographics		n (%)
Gender	Female	152 (67.6)
	Male	73 (32.4)
Age (years)	Mean (SD)	38.5 (15.5)
	Range	7-86
Occupation	Not employed/retired	57 (25.3)
	Housewife	48 (21.3)
	Employee	34 (15.1)
	Work in the health sector (nurse/doctor)	29 (12.9)
	Student	21 (9.3)
	Other	29 (12.9)
Marital status	Single	75 (33.3)
	Married	141 (62.7)
	Widow/divorced	9 (4.0)
Educational status	Uneducated	12 (5.3)
	Primary	31 (13.8)
	High School	83 (36.9)
	University	88 (39.1)
	Post-graduate	11 (4.9)
Source/place of answering the questionnaire	Staff clinic	21 (9.3)
	Out-patient department	204 (90.7)

Most participants were seen in the OPD (90.7%, n=204). Notably, 67.6% (n=152) had regular follow-up for chronic diseases, and 70.7% (n=159) had received health education advice from the hospital (Table 2).

**Table 2:** Patterns of healthcare engagement among participants The data is presented in frequency (n) and percentage (%).

Patterns of healthcare engagement		n (%)
Regular follow-up for chronic disease(s)?	No	63 (28.0)
	Yes	152 (67.6)
Received any health education advice from this hospital?	No	66 (29.3)
	Yes	159 (70.7)

**Figure 1:**
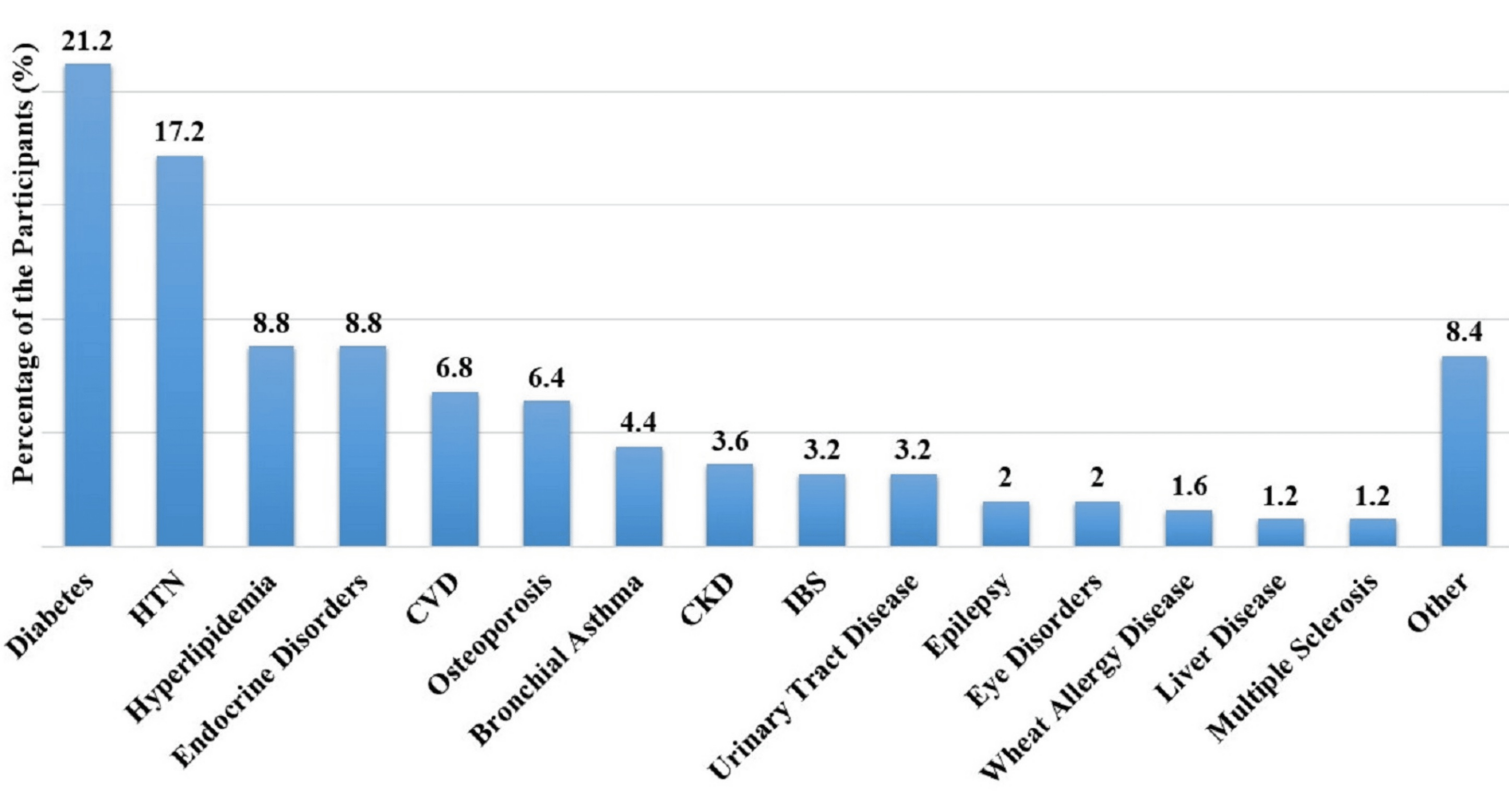
Different chronic diseases with which participants suffering (n=225) The data is presented as percentage (%). HTN: hypertension; CVD: cardiovascular diseases; CKD chronic kidney disease; IBS: irritable bowel syndrome

Diabetes (DM) was the most common condition, affecting 21.2% (n=48) of participants, followed by hypertension (HTN) at 17.2% (n=39). Hyperlipidemia and endocrine disorders other than DM each account for 8.8% (n=20). Cardiovascular disease (CVD) is reported by 6.8% (n=15), osteoporosis by 6.4% (n=14), and bronchial asthma by 4.4% (n=10). Less prevalent conditions included chronic kidney disease (CKD) at 3.6% (n=8), irritable bowel syndrome (IBS) and urinary tract disease at 3.2% (n=7), epilepsy and eye disorders at 2% (n=5) each. Wheat allergy disease, liver disease, and multiple sclerosis each had a prevalence of 1.6% (n=4), 1.2% (n=3), and 1.2% (n=3), respectively (Figure 1).

**Figure 2:**
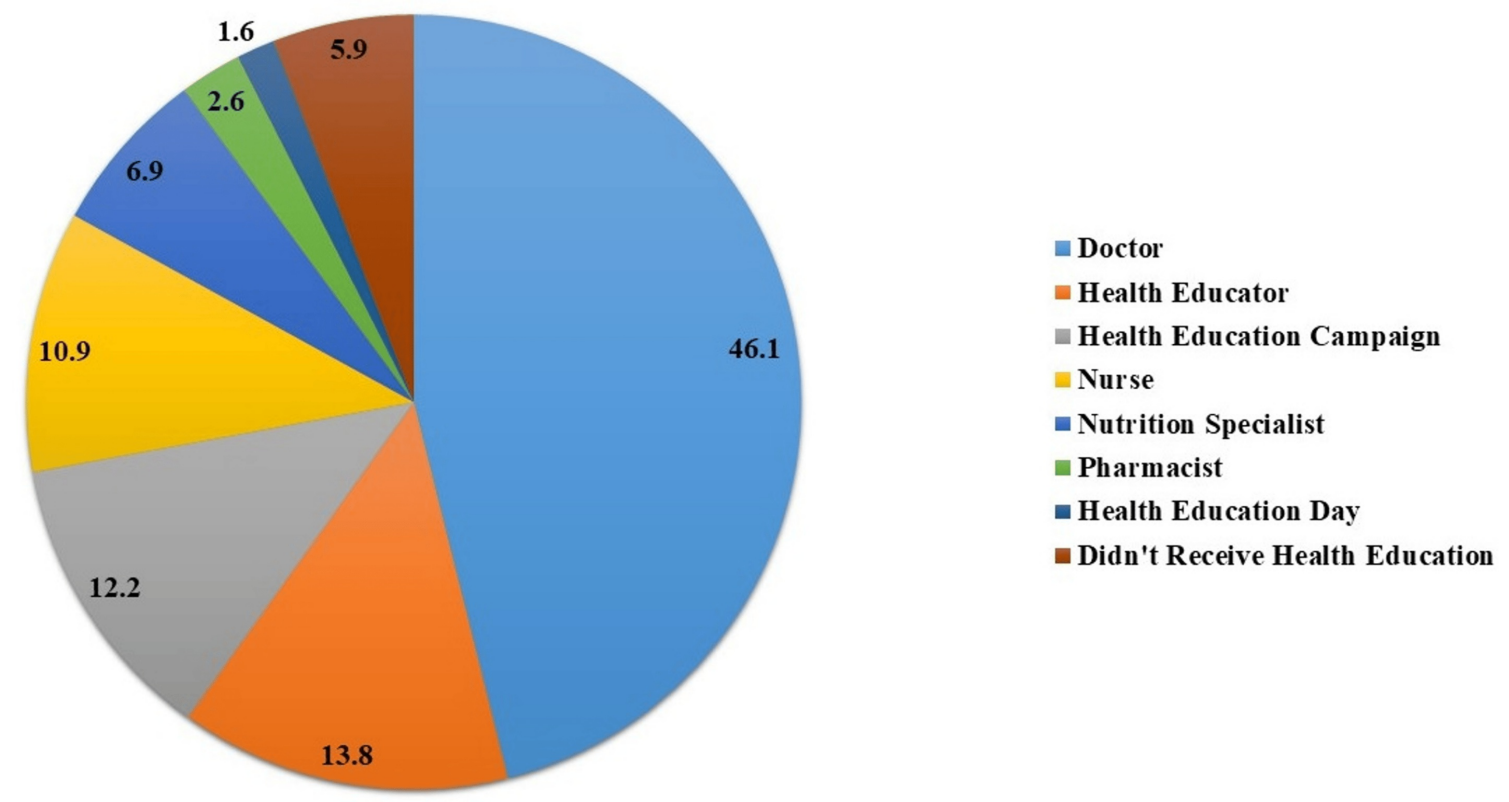
Source of health education advice for participants (n=225) The data is presented as percentage (%).

The majority (46.1%, n=104) received advice from doctors, while 13.8% (n=31) obtained information from health educators. Health education campaigns were informative for 12.2% (n=28), and nurses played a role for 10.9% (n=24). Nutrition specialists were a source for 6.9% (n=15), pharmacists helped 2.6% (n=6), and health education days were helpful for 1.6% (n=4). A notable 5.9% (n=14) reported not receiving health education (Figure 2).

The majority expressed agreement or strong agreement across various parameters based on the study questionnaire that had questions designed as a Likert scale with five subgroups. Notably, 80.4% (n=181) found the seating area comfortable, while 74.2% (n=167) agreed that the pre-session waiting time was sufficient. A significant proportion, 82.7% (n=186), reported a pleasant clinic atmosphere, and 75.5% (n=170) found the room well-furnished and spacious. Access to print materials was satisfactory for 70.7% (n=159), and 75.5% (n=170) found audiovisual materials readily available. Moreover, 76.0% (n=171) considered the allotted time for sessions sufficient, and 70.2% (n=158) found scheduling follow-ups easy (Table 3).

**Table 3:** Contentment regarding the structural aspects of health education service delivery (n=225) The data is presented in frequency (n) and percentage (%).

Structural aspects of health education service delivery	Strongly disagree	Disagree	Neutral	Agree	Strongly agree
	n (%)	n (%)	n (%)	n (%)	n (%)
The seating area provided a comfortable environment	2 (0.9)	15 (6.7)	27 (12.0)	93 (41.3)	88 (39.1)
The pre-session waiting time for the health educator was sufficient	7 (3.1)	17 (7.6)	34 (15.1)	103 (45.8)	64 (28.4)
The clinic's atmosphere was rather pleasant	5 (2.2)	5 (2.2)	29 (12.9)	101 (44.9)	85 (37.8)
The room was well-furnished and spacious	5 (2.2)	10 (4.4)	40 (17.8)	93 (41.3)	77 (34.2)
The print materials were reachable	8 (3.6)	12 (5.3)	46 (20.4)	96 (42.7)	63 (28.0)
The audiovisual materials were readily available	8 (3.6)	11 (4.9)	36 (16.0)	97 (43.1)	73 (32.4)
The allotted time for the health education session was sufficient	6 (2.7)	6 (2.7)	42 (18.7)	91 (40.4)	80 (35.6)
Scheduling a new appointment or follow-up with the health educator was easy	12 (5.3)	15 (6.7)	40 (17.8)	83 (36.9)	75 (33.3)

Notably, 88% (n=198) found the courtesy and care displayed to be exceptional, and 86.2% (n=194) felt their queries received perfect attention. Communication abilities were commendable for 81.8% (n=184), and 84.4% (n=190) perceived healthcare providers' knowledge and attitude as perfect. Moreover, 88.8% (n=200) reported that guidance was expressed in layman's terms, and 78.7% (n=177) actively participated in educational session planning (Table 4).

**Table 4:** Contentment with the healthcare provider delivering health education (process and outcomes) (n=225) The data is presented in frequency (n) and percentage (%).

Health education delivery	Strongly disagree	Disagree	Neutral	Agree	Strongly agree
	n (%)	n (%)	n (%)	n (%)	n (%)
The courtesy and care displayed were exceptional	5 (2.2)	5 (2.2)	17 (7.6)	105 (46.7)	93 (41.3)
The interest and attention to my queries were perfect	4 (1.8)	7 (3.1)	20 (8.9)	99 (44.0)	95 (42.2)
The communication abilities were commendable	4 (1.8)	7 (3.1)	30 (13.3)	99 (44.0)	85 (37.8)
Their knowledge and attitude were perfect	4 (1.8)	4 (1.8)	27 (12.0)	102 (45.3)	88 (39.1)
The guidance provided was expressed in layman's terms	4 (1.8)	6 (2.7)	15 (6.7)	109 (48.4)	91 (40.4)
I actively participated in the educational session planning and decision-making	7 (3.1)	11 (4.9)	30 (13.3)	102 (45.3)	75 (33.3)

Notably, 67.5% (n=152) found the materials valuable in managing their conditions, and 67.1% (n=151) retained and revisited the contents. A significant proportion, 66.2% (n=149), felt the materials provided consistent and valuable guidance. Additionally, 60.4% (n=136) shared the materials with family or friends facing similar conditions. Regarding recommendations, 69.8% (n=157) stated they would recommend the service, and 72.9% (n=164) rated their overall experience as excellent (Table 5).

**Table 5:** Reaction to health education print materials (both the process and outcomes) (n=225) The data is presented in frequency (n) and percentage (%). KSMC: King Saud Medical City

Characteristics of health education print material	Strongly disagree	Disagree	Neutral	Agree	Strongly agree
	n (%)	n (%)	n (%)	n (%)	n (%)
It was valuable in assisting me in managing my condition	16 (7.1)	7 (3.1)	50 (22.2)	86 (38.2)	66 (29.3)
I retained it and revisited its contents	17 (7.6)	8 (3.6)	49 (21.8)	91 (40.4)	60 (26.7)
It provided consistent and valuable guidance	16 (7.1)	10 (4.4)	50 (22.2)	84 (37.3)	65 (28.9)
I shared it with a family member or friend facing a similar condition	16 (7.1)	19 (8.4)	54 (24.0)	76 (33.8)	60 (26.7)
I will certainly recommend this service to my friends and family	17 (7.6)	8 (3.6)	43 (19.1)	81 (36.0)	76 (33.8)
I would rate my overall experience with the health education services at KSMC as excellent	17 (7.6)	6 (2.7)	38 (16.9)	82 (36.4)	82 (36.4)

The mean contentment scores (±SD) for structural aspects of health education service delivery, healthcare provider delivery, and health education print materials were 31.8 (±6.1), 24.9 (±4.7), and 22.7 (±6.4), respectively. The Friedman Test indicated a statistically significant difference in satisfaction scores across these aspects (p-value≤0.001). Participants showed the highest satisfaction with the structural aspects of service delivery, followed by healthcare provider delivery, and then health education print materials (Table 6).

**Table 6:** Satisfaction score between different aspects of health education The data is presented in mean and standard deviation (SD). ^a^: Friedman test

Aspects of health education	Mean contentment score (±SD)	^a^p-value
Contented with the structural aspects of health education service delivery	31.8 (6.1)	<0.001
Contented with the healthcare provider delivering health education	24.9 (4.7)	
Contented with health education print materials	22.7 (6.4)	

Among the notable findings, the occupation of individuals demonstrated a significantly higher odds ratio (aOR=1.498, p=0.048^*^) for contentment. Similarly, those with higher education levels exhibited lower odds of contentment (aOR=0.420, p=0.017^*^). Age showed a marginally insignificant but positive relationship with contentment (B=0.043, p=0.062). Interestingly, participants who had received health education before tended to have higher odds of contentment, although this relationship was not statistically significant (aOR=2.306, p=0.097) (Table 7).

**Table 7:** Adjusted sociodemographic predictors of patient contentment regarding health education aOR: adjusted odd’s ratio * denotes statistically significant p-values, with p < 0.05 considered significant

Adjusted sociodemographic predictors of patient contentment	Exponent B	Significance	Adjusted odds ratio (aOR)	95% confidence interval (CI)	
				Lower	Upper
Gender (male)	-0.325	0.553	0.723	0.248	2.111
Nationality (Saudi)	0.203	0.770	1.225	0.315	4.763
Age	0.043	0.062	1.044	0.998	1.091
Occupation	0.404	0.048^*^	1.498	1.003	2.238
Marital status (married)	-0.688	0.219	0.502	0.168	1.506
Higher education	-0.868	0.017^*^	0.420	0.206	0.854
Source of sample (outpatient department)	-0.030	0.971	0.971	0.197	4.781
Regular follow-up of chronic diseases (yes)	-0.620	0.270	0.538	0.179	1.620
Ever received health education (yes)	0.836	0.097	2.306	0.861	6.180
Constant	3.299	0.201	27.077		

ANOVA indicated a significant difference (p-value=0.002). Students scored highest (86.42±11.65), followed by housewives (83.2±11.88). Employees, health-sector workers, and unemployed individuals had progressively lower scores (Table 8).

**Table 8:** Patient contentment score regarding health education for occupation The data is presented in mean and standard deviation (SD). ^a^: ANOVA; higher score indicates higher contentment

Occupation	N	Mean (SD)	^a^p-value
Student	21	86.42 (±11.65)	0.002
Housewife	48	83.2 (±11.88)	
Employee	34	80.67 (±13.72)	
Health-sector worker	29	78.06 (±17.46)	
Unemployed	57	73.78 (±15.05)	
Total	189	79.48 (±14.66)	

## Discussion

Notably, the prevalence of chronic diseases among participants revealed DM as the most common condition (21.2%), followed by HTN (17.2%). These findings align with global trends, emphasizing the importance of health education in managing prevalent conditions like DM and HTN. Similarly, Al-Khaldi and Al-Sharif (2005) stated that health education aids treatment adherence and prevention through periodic examinations and screenings for prevalent conditions like DM and HTN [17]. Additionally, the study identified a range of less common diseases, highlighting the diversity of health concerns addressed in health education services.

There were diverse sources of health education advice, with doctors being the primary source (46.1%). This aligns with the traditional role of healthcare professionals as key educators. Moyoh et al. (2022) say that patient education has been traditionally seen as the responsibility of nurses and physicians [18]. Health educators and campaigns also played substantial roles, underscoring the multidisciplinary approach to health education services [19]. The 5.9% who did not receive health education suggested potential gaps that should be explored to ensure comprehensive coverage.

This study provided a detailed analysis of participants' contentment with the structural aspects of health education service delivery. The majority expressed high levels of satisfaction across various parameters, emphasizing the importance of a conducive environment, sufficient waiting times, and accessible educational materials. These findings resonate with existing literature emphasizing the significance of the physical and organizational aspects of healthcare settings in enhancing patient satisfaction. Similarly, Ferreira et al. (2023) identified nine determinants of satisfaction including technical skills, interpersonal care, physical environment, accessibility, availability, finances, organizational characteristics, continuity of care, and care outcome [20].

Similarly, regarding the contentment with healthcare providers delivering health education, the high satisfaction levels with courtesy, care, attention to queries, communication abilities, and guidance align with the pivotal role healthcare providers' play in effective health education. These findings highlighted the importance of interpersonal skills and effective communication in delivering health education. Similarly, Chichirez and Purcărea (2018) showed that competent communication was crucial in establishing trust and fostering a therapeutic alliance between medical staff and patients [21].

Moreover, the positive reactions to health education print materials, emphasize their value in managing conditions and providing consistent guidance. The high percentage of participants sharing materials with others underscores their perceived utility. These findings align with the literature emphasizing the effectiveness of educational materials in empowering patients and promoting self-management of health conditions. Similarly, Bhattad and Pacifico (2022) show that patient education materials enhance health literacy, inform decision-making, and empower patients with current medical evidence and preferences [22].

The satisfaction scores across different aspects of health education indicate the highest satisfaction with the structural aspects of service delivery, followed by healthcare provider delivery and health education print materials. The significant difference in scores emphasizes the need for a holistic approach, addressing both physical and interpersonal elements to enhance overall satisfaction. Ambushe et al. (2023) showed that holistic nursing care is an approach to patient care that takes into account the physical, social, spiritual, and psychological needs of the patient [23].

Moreover, the logistic regression model explored sociodemographic predictors of patient contentment. Occupation and education level emerged as significant predictors, emphasizing the influence of these factors on satisfaction. Similarly, Tateke et al. (2012) showed that occupational and educational status had a statistically significant association with the patient satisfaction score [24]. In contrast with the current study, Afzal (2012) showed that patient satisfaction rises with higher education levels, evident in increased rates and mean satisfaction scores [25]. Tailoring health education strategies to different occupational and educational backgrounds could enhance overall contentment.

While the study provides valuable insights, certain limitations should be acknowledged. The study included 225 participants, which might not be representative of the entire patient population at KSMC. The predominance of female participants (67.6%) might also skew the results. The study's cross-sectional nature provides a snapshot of patient satisfaction at a single point in time, making it difficult to determine causal relationships or long-term trends. Data were collected through self-reported questionnaires, which can introduce bias due to participants' perceptions and memory recall. Conducting the study at a single medical facility limits the generalizability of the findings to other settings or regions.

Future studies should include larger and more diverse samples to improve the generalizability of the findings. Including a balanced gender representation and a wider range of health conditions would provide a more comprehensive understanding. Conducting longitudinal studies could help identify changes in patient satisfaction over time and assess the long-term impact of health education interventions. Expanding the study to multiple healthcare facilities across different regions would enhance the applicability of the findings and allow for comparisons between different settings. Including qualitative methods such as interviews or focus groups could provide deeper insights into patient experiences and perceptions, complementing the quantitative data. Developing tailored health education strategies based on occupation and education levels, as indicated by the study, could improve patient satisfaction and outcomes. Future research should evaluate the effectiveness of these customized approaches.

## Conclusions

This study contributed significantly to understanding patient contentment with health education services at KSMC. The findings emphasize the importance of a comprehensive, patient-centered approach, considering both structural and interpersonal aspects. Tailoring interventions to demographic characteristics could further enhance the effectiveness of health education strategies, promote patient empowerment, and improve health outcomes.
